# Comprehensive device simulation of 23.36% efficient two-terminal perovskite-PbS CQD tandem solar cell for low-cost applications

**DOI:** 10.1038/s41598-021-99098-y

**Published:** 2021-10-06

**Authors:** Jaya Madan, Karanveer Singh, Rahul Pandey

**Affiliations:** 1 VLSI Centre of Excellence, Chitkara University Institute of Engineering and Technology, Chitkara University, Rajpura, Punjab India; 2 Chitkara College of Applied Engineering, Chitkara University, Rajpura, Punjab India

**Keywords:** Solar cells, Electronics, photonics and device physics

## Abstract

The major losses that limit the efficiency of a single-junction solar cell are thermalization loss and transmission loss. Thus, to efficiently utilize the full solar spectrum and to mitigate these losses, tandem solar cells (TSC) have significantly impacted the photovoltaic (PV) landscape. In this context, the research on perovskite/silicon tandems is currently dominating the research community. The stability improvements of perovskite materials and mature fabrication techniques of silicon have underpinned the rapid progress of perovskite/silicon TSC. However, the low absorption coefficient and high module cost of the silicon are the tailbacks for the mass production of perovskite/silicon TSCs. Therefore, PV technology demands to explore some new materials other than Si to be used as absorber layer in the bottom cell. Thus, here in this work, to mitigate the aforementioned losses and to reduce cost, a 23.36% efficient two-terminal perovskite-PbS CQD monolithic tandem solar cell has been designed through comprehensive device simulations. Before analyzing the performance of the proposed TSC, the performance of perovskite top cells has been optimized in terms of variation in optical properties, thickness, and interface defect density under standalone conditions. Thereafter, filtered spectrum and associated integrated filtered power by the top cell at different perovskite thickness from 50 to 500 nm is obtained to conceive the presence of the top cell above the bottom cell with different perovskite thickness. The current matching by concurrently varying the thickness of both the top and bottom subcell has also been done to obtain the maximum deliverable tandem J_SC_ for the device under consideration. The top/bottom subcell with current matched J_SC_ of 16.68 mA cm^−2^/16.62 mA cm^−2^ showed the conversion efficiency of 14.60%/9.07% under tandem configuration with an optimized thickness of 143 nm/470 nm, where the top cell is simulated under AM1.5G spectrum, and bottom cell is exposed to the spectrum filtered by 143 nm thick top cell. Further, the voltages at equal current points are added together to generate tandem J–V characteristics. This work concludes a 23.36% efficient perovskite-PbS CQD tandem design with 1.79 V (V_OC_), 16.67 mA cm^−2^ (J_SC_) and 78.3% (FF). The perovskite-PbS CQD tandem device proposed in this work may pave the way for the development of high-efficiency tandem solar cells for low-cost applications.

## Introduction

**Figure 1:**
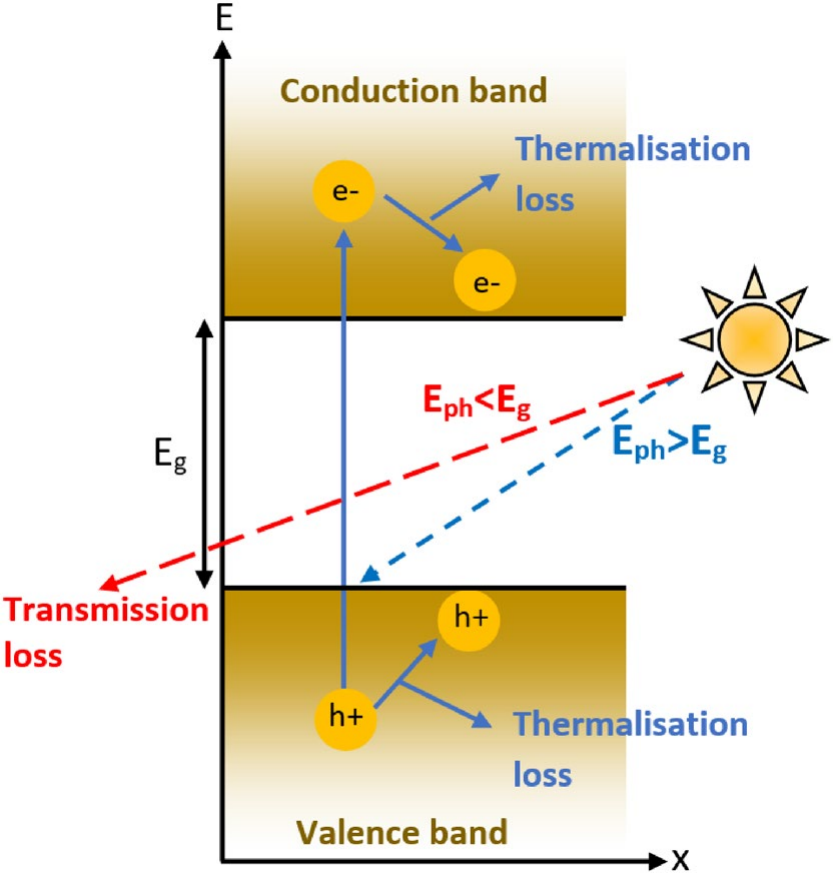
Schematic representation of power losses in single-junction solar cells viz. thermalization and transmission loss.

To achieve the golden triangle elements (efficiency, cost, and lifetime), the researchers are extensively working on different semiconductor materials to develop high-efficiency solar cells. However, the single-junction solar cell suffers mainly from two inherent major power loss mechanisms, viz. thermalization and transmission (transparent E_g_ or non-absorbed photon) losses, depicted pictorially in Fig. 1. It shows that the high-energy photons (low wavelengths), in comparison with the bandgap of the material, excites the electron residing deep inside the valence band towards deep inside the conduction band. Thereafter, the electron (hole) generated deep inside the conduction (valence) band loses their energy as heat to relax to conduction (valence) band edges^1^. This loss of the incoming solar energy is known as thermalization loss. While, for the low-energy photons (higher wavelength) in comparison with the bandgap of the material, the photon will not be absorbed by the material and will not participate in the generation of charge carriers. This is known as the transmission loss or non-absorbed photon loss^1^. Additionally, to surpass the Shockley-Queisser single-junction limits^2^, abundant trails were made to fabricate solar cells with multiple junctions. i.e., tandem solar cells^3–9^. Tandem cells tackle with both thermalizations as well as transmission photon loss, which contributes mainly in the reduction of PCE of single-junction solar cells.

Tandem cells utilize the concept of dividing the power spectrum among multiple absorbers. The absorbers are selected in such a way that one of them is responsible for absorbing lower wavelength photons, while the other is assigned for photons of higher wavelength. With optimized bandgaps, a two-cell stacked tandem configuration can achieve up to 42% PCE, as stated in literature^3^. Further, in the year 1996, *Martí *et al*.* gave a hypothetical statement that under the standard AM1.5 illumination spectrum, an infinite stack of junctions can yield up to 65% PCE^10^. Typically, a two-cell stacked tandem configuration consists of a wide bandgap top cell and a narrow bandgap bottom cell to utilize high and low energy photons, respectively. Thereby the top cell reduces the transmission loss of the bottom cell while the bottom cell reduces the thermalization losses of top cell and utilizes the sun spectrum more efficiently.

For tandem cells to have better performance, researchers have used different combinations of sub-cells which include perovskite/perovskite^7,11–14^, perovskite/silicon^8,15–18^, perovskite/CIGS^19,20^, and all quantum dot^20,21^ structure and many more. All these combinations had performed well and had seen a massive rise in the performance over the decade. In most of the above-mentioned tandem configuration, perovskite, with its tunable bandgap (1.1 to 2.3 eV) and low processing cost techniques, emerged as developing material for standalone as well as tandem PV technology. The tremendous research effort of the researchers across the globe resulted in a recent record conversion efficiency of 25.6%^22^ followed by previous record efficiency of 25.2%^23,24^. The enhancement is significant compared to the initial value of conversion efficiency 3.8%, as reported in the year 2009 ^25^. The perovskite-silicon tandem received great attention from the researchers that led to different fabrication routes for the perovskite top cell over silicon-based bottom subcells such as spin coating^6^, two-step hybrid method^26,27^, blade coating^28^, and slot-die coating^29^. Except for spin coating, the rest of the methods are investigated so as to provide a perovskite-silicon tandem design with a technique feasible at the industrial level. As of now, perovskite-silicon tandem solar cell reflected a record conversion efficiency of 29.5%^30^ which followed by an efficiency of 29.16%^6^, and the battle is still on for further improvement. However, there are some innate problems associated with perovskite-silicon tandem devices, such as low absorption coefficient and high module cost, which are the tailback for its mass production at the industrial level^7,9,31–33^.

Therefore, considering the detailed balance limit for the tandem solar cell, a top cell with 1.55 eV and a low-cost solution-processed bottom cell with a tunable bandgap can be well thought for the low-cost tandem design. This gives the track to the semiconductor materials that can be processed at a reduced cost to demonstrate their potential in tandem with perovskite top cells. Lead (II) sulfide colloidal quantum dots (PbS CQDs) are appropriate materials for constructing such complex multi-junction architectures because of their exceptional processibility and brilliant photoelectric properties. The conventional layer-by-layer deposition method provides them with benefits in the fabrication of tandem structures. Apart from this, tunable bandgaps can be achieved for a broad range of energies by varying the average QD size. Furthermore, in ambient conditions, CQDs offer low-cost solution processing methods and also provide additional absorption in the infrared (IR) region, which extends the absorption edge further to harvest low-energy photons in tandem configuration. These valuable properties resulted in exploring the potential of PbS CQDs-based bottom cell in the tandem configuration^9,34–37^.

Now, considering close to ideal properties of perovskite-based top cell and PbS CQDs-based bottom cell for tandem configuration. A perovskite-PbS CQDs-based two-terminal monolithic tandem solar cell has been designed, and detailed investigation and optimization have been performed to realize 23.36% efficient perovskite-PbS CQDs tandem solar cell. In the tandem configuration, the performance of the bottom cell highly depends on the transmitted spectrum by the top cell. Therefore, optical properties of the top cell play a vital role in determining the performance of the bottom cell and overall tandem performance. In literature, the optical properties of perovskite represented huge variations in different scholarly articles^38–42^, which is not in the case of any other semiconductor material. The consequences of such variations on the performance of perovskite solar cells (PSCs) have not been disclosed previously. Therefore, a detailed investigation of standalone perovskite top cell is done with different optical properties as reported in pieces of literature^38–42^, and the optimized top cell is sent forward for the tandem configuration with the PbS CQDs based bottom cell.

The overall manuscript is divided into four subsections. The ongoing introduction section is followed by the device structure and simulation methodology section, which provides a thorough discussion related to simulation methods, material parameters, and filtered spectrum. Thereafter, the result and discussion section are provided, which reveals the influence of the variation in the absorption coefficient of perovskite, perovskite thickness, and perovskite/ETL interface defect density followed by a design of 20.57% perovskite-PbS CQD tandem solar cell. The summary of the research work is concluded in the conclusion section, along with the future scope of the work.

## Device structure and simulation methodology

**Figure 2:**
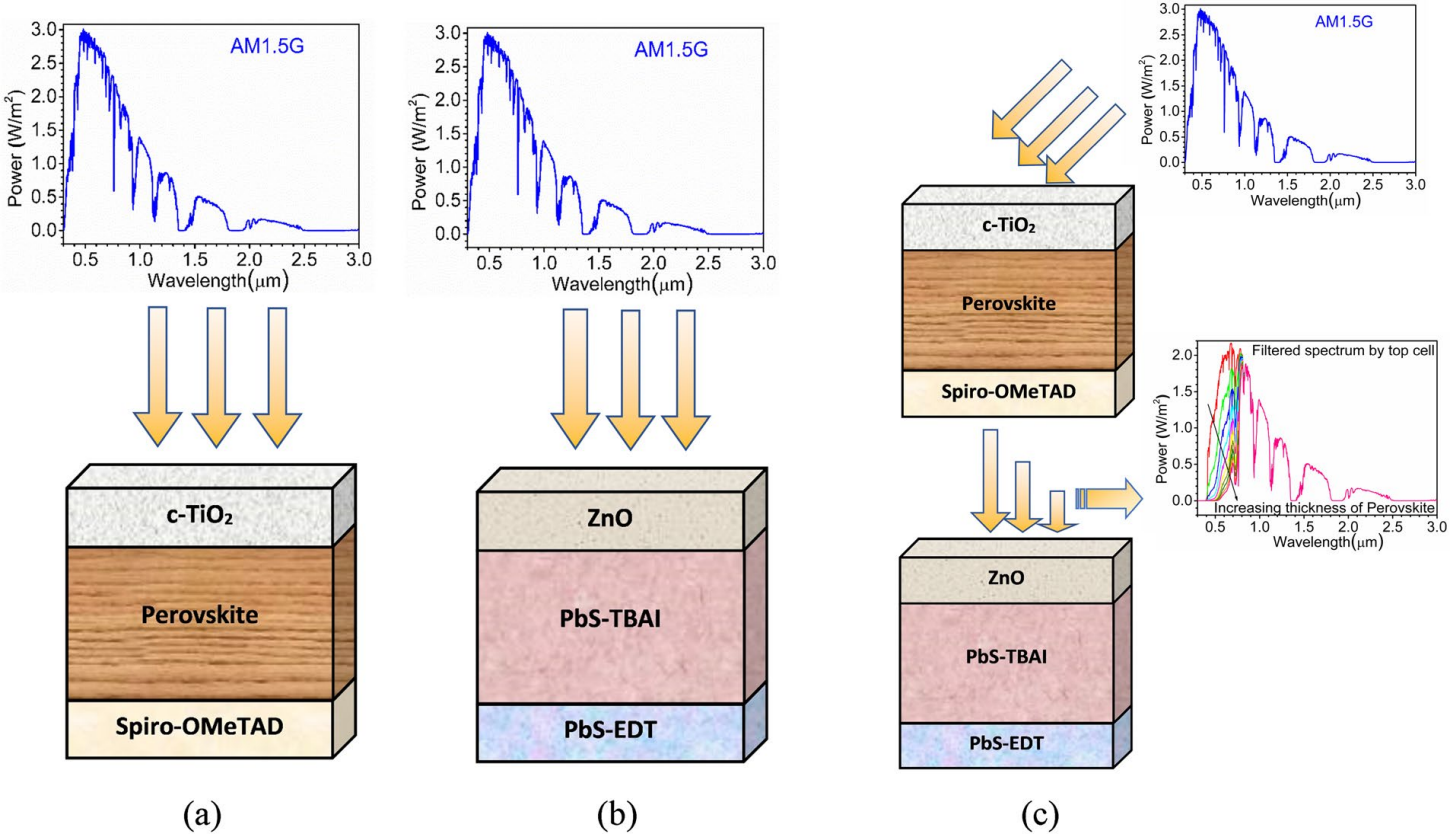
Simulated device structures along with the illuminated spectrum (**a**) perovskite cell under standalone condition (**b**) PbS-CQD cell under standalone condition (**c**) tandem solar cell with perovskite as the top cell (illuminated with standard AM1.5G) and PbS-CQD as the bottom cell (illuminated with the filtered spectrum of the top cell). The optical filter file for ITO has been applied to the electrode facing the illumination under both standalone and tandem configurations.

In this work, a tandem solar cell with perovskite (CH_3_NH_3_PbI_3_) as the top cell and PbS CQDs as the bottom cell has been designed using the SCAPS-1D simulator (a Solar Cell Capacitance Simulator). SCAPS, an open-source tool for designing solar cells, is developed at the department of electronics and information systems (ELIS), University of Ghent, Belgium^43^. Before simulating the proposed tandem perovskite-PbS CQDs design, both top and bottom cells are examined under standalone conditions, i.e., with AM1.5G spectrum illumination. The top cell utilized 1.55 eV perovskite (325 nm) as an absorber layer along with TiO_2_ (90 nm) as electron extraction layer (ETL) and Spiro-OMeTAD (460 nm) as hole extraction layer (HTL) depicted in Fig. 2(a). While the bottom cell comprises of a low bandgap absorber material, i.e., PbS- CQD layer treated tetrabutylammonium iodide (PbS-TBAI) (230 nm) with a bandgap of 1.14 eV having Mg-doped ZnO (MZO) (80 nm) as ETL and PbS CQD treated with 1, 2-ethanedithiol (PbS-EDT) (50 nm) as HTL as shown in Fig. 2(b). Both top and bottom cells are calibrated as per experimental work, and the calibration details for the top cell is provided in the results and discussion section, whereas MZO ETL based bottom cell is calibrated as per the device design published by Zhang et al*.*^44^ to have 9.43% efficient bottom cell. The results of the finding are published elsewhere^45^, and the same optimized device is invoked to be used as the bottom subcell.

Afterward, both top and bottom subcells were stacked one over the other to form a monolithically integrated tandem device with perovskite-based wide-bandgap sub-cell as top cell and the PbS-CQDs based narrow bandgap sub-cell as bottom cell as shown in Fig. 2(c). The appropriate gap between the bandgaps of the two sub-cells makes them ideal for tandem structure. In two-terminal tandem solar cells stacking of two sub-cells results in the formation of a reverse-biased *p–n* junction at the interface of two subcells which eventually restricts current flow across the device^46^. To overcome this issue, the current matching strategy is utilized where a tunnel recombination junction (TRJ) is used, which ensures suitable equal current flow across both the devices in tandem configuration. However, owing to the limitations of SCAPS 1-D software, such TRJ is not feasible, and simulation of tandem structure is quite cumbersome. Therefore, an ideal TRJ layer is assumed without optoelectronic losses, where both top and bottom subcells are simulated independently but with different illumination spectrums. The top cell is illuminated with the standard AM1.5G spectrum while the transmitted filtered spectrum of the top cell is fed to the bottom cell so as to derive essential current matching conditions followed by construction of tandem current density–voltage (J–V) curve. This approach is widely adopted by the researchers designing tandem solar cells using SCAPS-1D^7,46–49^, and detailed information for calculating the absorbed and transmitted photocurrent along with filtered spectrum by the top cell at various thicknesses is provided in one of our previous publication^7^. The following formula is used to calculate the filtered spectrum by top cell at different absorber layer thicknesses.

$$ S(\lambda ) = S_{0} (\lambda ) \cdot \exp \left( {\sum\limits_{i = 1}^{3} { - \left( {\alpha_{{material_{i} }} (\lambda ) \cdot d_{{material_{i} }} } \right)} } \right) $$

Here, *S*_*o*_(*λ*) is the standard AM1.5G spectrum*, S(λ)* is the transmitted spectrum by the top cell, *α(λ)* is the absorption coefficient, and *d* is the thickness of the respective layers. The *material*_*1*_, *material*_*2*_*,* and *material*_*3*_ represent c-TiO_2_, perovskite, and Spiro-OMeTAD layers, respectively. The interfacial reflection losses are ignored while computing the filtered spectrum. The extensive literature survey is done to obtain the electrical as well as optical properties for all the materials used during the simulation, and the obtained electrical parameters are reported in Table 1. The interfacial defects at the heterojunctions are also considered, and information is provided in Table 2. The optical properties of the bottom cell are obtained from^44^ whereas details regarding the optical properties of the top cell are provided in the results section. SCAPS-1D numerically solves coupled Poisson’s and continuity equations with dedicated boundary conditions of both holes and electrons for different interfaces and contacts. The fundamental equations for the optical model of SCAPS-1D and semiconductor equations used to simulate the optoelectronic performance are in accordance with the previously published work^7^, and reiteration of the same is avoided in this work. The fundamental monomolecular, bimolecular and trimolecular recombination rate in the perovskite is also considered in accordance with already published work^17,50,51^.

**Table 1:** Electrical properties of materials used in top cell, bottom cell, and tandem solar cell^8,9,17,32–34,44,45,52^.

Parameter	Material					
	TiO_2_	Perovskite	Spiro-OMeTAD	MgZnO (MZO)	PbS-TBAI	PbS-EDT
Bandgap (eV)	3.20	1.55	3.00	3.35	1.14	1.14
Electron affinity (eV)	3.9	3.9	2.45	4.0	4.0	3.9
Dielectric Permittivity	9	6.5	3	66	20	20
CB effective density of states (cm^−3^)	2.2 × 10^18^	2.2 × 10^18^	2.2 × 10^18^	1 × 10^19^	1 × 10^19^	1 × 10^19^
VB effective density of states (cm^−3^)	1.8 × 10^19^	1.8 × 10^19^	1.8 × 10^19^	1 × 10^19^	1 × 10^19^	1 × 10^19^
Electron mobility (cm^2^V^−1^ s^−1^)	20	2	2.0 × 10^−4^	5.0 × 10^−2^	2.0 × 10^−2^	2.0 × 10^−4^
Hole mobility (cm^2^V^−1^ s^−1^)	10	2	2.0 × 10^−4^	5.0 × 10^−2^	2.0 × 10^−2^	2.0 × 10^−4^
Donor density N_D_ (cm^−3^)	1 × 10^16^	0	0	1 × 10^17^	1 × 10^15^	1 × 10^14^
Acceptor density N_A_ (cm^−3^)	0	1 × 10^14^	1 × 10^18^	0	1 × 10^15^	1 × 10^16^

The second and third-order recombination rates are also considered for the perovskite with the help of charge carrier decay constants, as reported in previous literature ^17,50,51^.

**Table 2:** Interface defects are considered at different interfaces of the top cell, bottom cell, and tandem solar cell.

Parameters	Interface			
	PbS-EDT/ PbS-TBAI interface	PbS-TBAI/ MZO interface	Spiro-OMeTAD/ Perovskite interface	Perovskite/ TiO_2_ interface
Capture cross section electrons (cm^2^)	1.2 × 10^−13^	1.0 × 10^−19^	1.0 × 10^−15^	1.0 × 10^−15^
Capture cross section holes (cm^2^)	1.2 × 10^−13^	1.0 × 10^−19^	1.0 × 10^−15^	1.0 × 10^−15^
Energy with respect to reference (eV)	0.50	0.60	0.75	0.75
Total density (integrated over all energies) (cm^−2^)	1 × 10^16^	2 × 10^14^	1 × 10^11^	1 × 10^11^

For all the cases, the defect type is neutral with the single energetic distribution. The reference for defect energy level (E_t_) is above the highest E_V_.

For neutral defects, carrier lifetimes are determined using $\tau_{n} = 1/\left( {\sigma_{n} v_{th} N_{t} } \right)$ and $\tau_{p} = 1/\left( {\sigma_{p} v_{th} N_{t} } \right)$. Where $\sigma_{n} , \, \sigma_{p} , \, v_{th \, } ,N_{t}$ are the electron capture cross-section, hole capture cross-section, thermal velocity (10^7^ cm/s), and total defect density, respectively.

## Results and discussion

### Influence of variation in the absorption coefficient of perovskite

In this initial part of the result section, a perovskite-based solar cell is designed using a SCAPS-1D simulator with 460 nm thick Spiro-OMeTAD as HTL, a 325 nm absorber layer of CH_3_NH_3_PbI_3_ and TiO_2_ as ETL with the thickness of 90 nm. After that, the effect of the deviation in the absorption coefficient of the perovskite material is studied. The optical properties were taken from the previous publications, which report different values of the real and imaginary parts of the refractive index, i.e., *n* and *k*, respectively. This initiative is taken because the variations in published articles are quite significant, which is not the same for general semiconductors. There could be many reasons behind this, as different researchers choose different ways of fabricating the device. Even adopting a similar procedure and with similar chemical composition cannot ensure similar grain size distribution, and hence, the extracted properties may scatter over a wide range of values. Some minor alterations can be caused by material anisotropy and the accuracy of property extraction techniques. The consequences of these variations are not considered in any of the theoretical work related to PSC to the best of our knowledge. Since previously reported works on PSC considered a fixed wavelength-dependent absorption coefficient and did not discuss the influence of variation in optical properties, particularly absorption coefficient (α = 4πk/λ).

**Figure 3:**
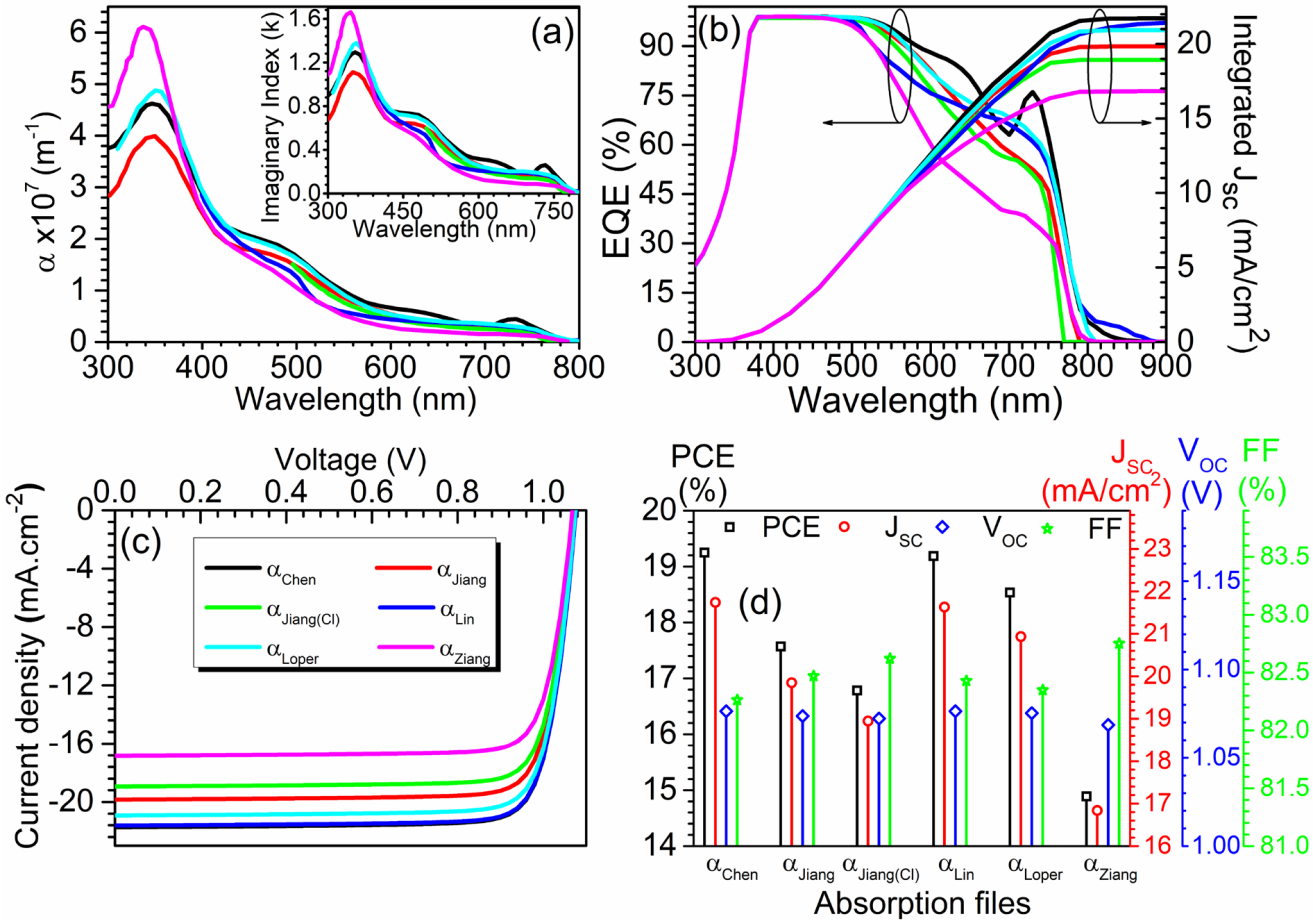
(**a**) Calculated room temperature absorption coefficient data for CH_3_NH_3_PbI_3_ and CH_3_NH_3_PbI_3_(Cl). Inset shows published room temperature *k* values (Data sources are Chen et al.^38^, Jiang et al.^39^, Lin et al.^40^, Löper et al.^41^, and Ziang et al.^42^ and Green et al.^53^ (**b**) EQE and integrated J_SC_, (**c**) illuminated J–V curve and (**d**) PV parameters such as J_SC_, V_OC_, FF, and PCE with different optical absorption files.

Therefore, the need to study such influence is perceived in this work. To study these variations, we calculated absorption coefficients and created absorption coefficient files (for the SCAPS-1D simulations) with the help of the imaginary parts of the refractive index (*k*) from five different sources such as *Chen *et al*.*^38^, *Jiang *et al*.*^39^, *Lin *et al*.*^40^, *Löper *et al*.*^41^, and *Ziang *et al*.*^42^. Calculated wavelength-dependent absorption coefficients from *Chen *et al*.*^38^, *Jiang *et al*.*, *Lin *et al*.*^40^, *Löper *et al*.*^41^, and *Ziang *et al*.*^42^ are termed as *α*_*Chen*_*, α*_*Jiang*_*, α*_*Jiang(Cl)*_*, α*_*Lin*_*, α*_*Löper*_*,* and *α*_*Ziang*_ and are shown in Fig. 3(a), the corresponding *k* values are also shown in the inset of the same figure. The *k* values corresponding to these pieces of literature are obtained from a single source, *Green *et al*.*^53^. All the generating absorption coefficient files are supplied to the simulator using batch setup while keeping the rest of the material parameters intact, and corresponding J–V and EQE with integrated J_SC_ curves are obtained along with PV parameters such as J_SC_, V_OC_, FF, and PCE as reported in Fig. 3(b-d). The EQE and integrated J_SC_ showed in Fig. 3(b) show the gradual difference in device performance, particularly in the 500–800 nm wavelength range. The integrated J_SC_ changes to 21.7 mA cm^−2^* → *19.8 mA cm^−2^ → 18.9 mA cm^−2^ → 21.4 mA cm^−2^ → 20.9 mA cm^−2^ → 16.8 mA cm^−2^ while changing the optical data from *α*_*Chen*_* → α*_*Jiang*_* → α*_*Jiang (Cl)*_* → α*_*Lin*_* → α*_*Löper*_* → α*_*Ziang*_. Result showed great variations, ranging from 16.8 mA cm^−2^ to 21.7 mA cm^−2^ for the optical data *α*_*Ziang*_ and *α*_*Chen*_, respectively. Change in absorption coefficient reflected change in optical absorption and corresponding EQE and integrated J_SC_ response. Figure 3(b) shows that device with superior optical response reflected highest J_SC_ of 21.7 mA cm^−2^.

Figure 3(c) and (d) are also produced to further quantify the rest of the PV parameters during the variation of the absorption file. The intersect with the current density axis and corresponding J_SC_ data reported in Fig. 3(d) are also in accordance with the integrated J_SC_ reported in Fig. 3(b) for the respective optical data. Results also reflected insignificant variation in V_OC_ and FF in the range of (1.076 V – 1.075 V) and (82.26% – 82.75%) with said variation in optical data. The collective influence of J_SC_, V_OC_, and FF are reflected in the PCE as shown in Fig. 3(d), which ranges from (14.88% – 19.25%) with the highest conversion efficiency of 19.25% with *α*_*Chen*_, followed by 19.19% with *α*_*Lin*_*.*

The validity of the simulated results with experimental findings is an important part of any theoretical work. Therefore, the range of obtained efficiency is compared with the published experimental data for PSCs from the year 2013 to 2015 with device architecture TiO_2_ (ETL)/ perovskite (absorber layer)/ Spiro-OMeTAD (HTL). The best research cell efficiency chart published by National Renewable Energy Laboratory (NREL) showed that the conversion efficiency for the PSCs during the year 2013–2015 was ranging around (13.8% to 19.8%)^54^, and these values are very close to the simulated efficiency range (14.88% – 19.25%) achieved in this work. This proves the accuracy and authenticity of the simulation study carried out in this manuscript. The comparison is deliberately restricted till the year 2015 since all the absorption coefficient data used during simulations are obtained from the kinds of literature published in the single year 2015. Therefore, material and device-engineered PSCs, including the record conversion efficiency (25.2%) published after 2015^24,55^, are ignored to have a fair comparison.

This ongoing subsection summarizes the impact of deviation in absorption coefficient on the performance of PSCs, and the next subsection discusses the influence of absorber layer thickness on the PV performance. All the analysis reported in subsequent subsections utilizes the optical file (*α*_*Chen*_).

### Impact of perovskite thickness

**Figure 4:**
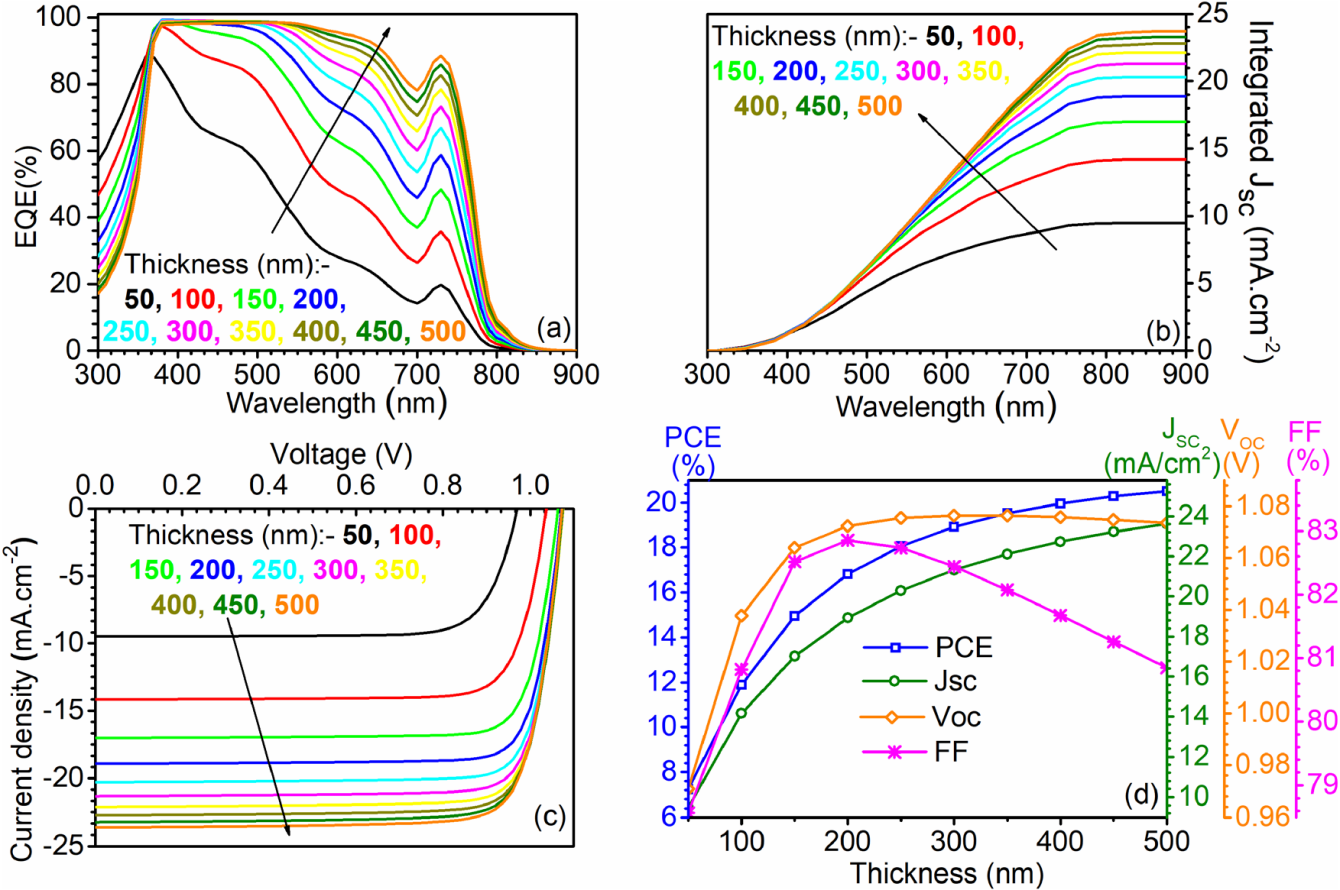
Impact of perovskite absorber layer thickness on (**a**) EQE, (**b**) integrated J_SC_, (**c**) illuminated J–V curve, and (**d**) PV parameters at various thicknesses, i.e., from 50 to 500 nm. Data reported in figures are obtained from the SCAPS-1D simulator.

The deposition of perovskite layer in sandwiched architecture-based PSCs mostly depends on the solution process-based spin coating techniques^56–58^, a standard method to deposit thin films with the thickness of a few nanometers to few microns. The entire process is divided into four main steps: deposition, spin-up, spin-off, and evaporation. The thickness of the perovskite absorber layer during the fabrication process of PSCs depends on several factors such as the concentration of the material and evaporation of the solvent, which further includes ambient humidity, temperature, vapour pressure, and viscosity of the solvent. Therefore, it is crucial to estimate the device performance with different thicknesses of the absorber layer. Hence, in this subsection, the analysis of the perovskite device has been made, where the influence of CH_3_NH_3_PbI_3_ thickness on the PV parameters is evaluated. The variation has been performed from 0.05 µm to 0.5 µm in ten equal steps in a linear scale, keeping the remaining device parameters intact. Corresponding EQE, integrated J_SC_, illuminated J–V curve, and PV parameters are summarized in Fig. 4(a), (b), (c), and (d), respectively.

It is recorded that increasing the thickness of perovskite resulted in an enhancement in the optical absorption and corresponding collection efficiency, particularly at higher wavelengths, as shown in Fig. 4(a). Since most of the lower wavelength photons tend to get absorbed in a small thickness and to absorb the higher wavelength photons, a relatively higher thickness is required to prevent the transmittance losses. Therefore, an increase in thickness resulted in lower transmittance loss, enhanced absorption, and collection probability at higher wavelengths. Quantitively, while increasing the thickness from 50 nm → 100 nm → 150 nm → 200 nm → 250 nm → 300 nm → 350 nm → 400 nm → 450 nm → 500 nm resulted in enhancement in EQE from 28.23% → 48.46% → 62.96% → 73.34% → 80.57% → 86.03% → 89.77% → 92.40% → 94.24% → 95.51% at a wavelength equal to 600 nm. Although increasing the thickness increases the optical performance, absorption saturation is a common phenomenon that restricts the improvement and limits the performance at higher thickness levels. The same is being reflected in this work also where the improvement in EQE beyond 350 nm thickness is not significant, as shown in Fig. 4(a).

Enhanced optical performance anticipate the improvement in integrated J_SC_, i.e., increasing the thickness from 50 to 350 nm resulted in J_SC_ improvement from 9.47 mA cm^−2^ to 22.10 mA cm^−2^ whereas further increasing the thickness beyond 350 nm to 500 nm resulted in 23.70 mA cm^−2^. Up to this point, only the influence on J_SC_ being examined whereas the overall conversion efficiency is the collective outcome of all three parameters such as J_SC_, V_OC_, and FF. Therefore, illuminated J–V curve and PV parameters are also summarized in Fig. 4(c) and (d), which shows that the V_OC_ also increases up to 250 nm and then indicates saturation. Improvement in V_OC_ is attributed to elevated concentration of light-generated charge carriers. Obtained FF also shows improvement until 200 nm due to an increase in carrier concentration. However, it started to fall while increasing the thickness beyond 200 nm due to reduced electric field strength across the perovskite layer. In case of the thick absorber layer, the collecting ETL/perovskite and perovskite/HTL interfaces move apart, and the associated electric field strength decreases. Therefore, as reported in Fig. 4 (d), conversion efficiency reflects improvement till 350 nm, and after that, marginal improvement is noticed.

This subsection concludes a PSC with a conversion efficiency of 19.52% at the perovskite thickness of 350 nm. The same device is invoked, and the influence of perovskite/ETL defect density on PV parameters is investigated in the following subsection.

### Impact of perovskite/transport layer defect density

**Figure 5:**
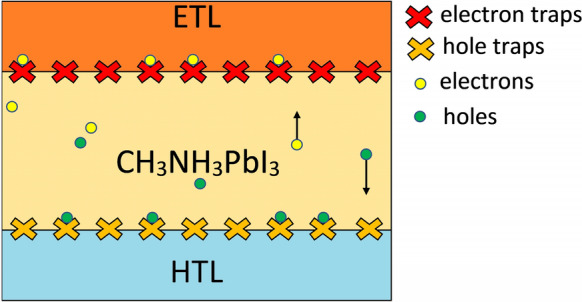
Problems associated with the interface of perovskite absorber layer/ETL and perovskite absorber layer/HTL.

In conventional sandwiched architecture-based PSCs, perovskite is sandwiched between ETL and HTL that consists of dissimilar semiconductor material with different electrical as well as structural properties compared to perovskite. The presence of transport layers helps in the extraction of light-generated carriers and provides higher conversion efficiencies. However, their presence also results in performance challenges in the form of interfacial recombination due to interface traps, hysteresis, and stability. The origin of interfacial traps related issues is projected in Fig. 5. The mismatch of electrical as well as structural properties between two different materials develops interfacial defects, which eventually results in elevated charge recombination at the interface, i.e., increased surface recombination velocity (SRV). Both perovskite/ETL and perovskite/HTL interface defects can significantly reduce the performance of PSCs. However, if illuminated from the ETL side, the impact of the perovskite/ETL interface defect would be large compared to the perovskite/HTL interface since most of the lower wavelength photons get absorbed in the vicinity of the perovskite/ETL interface and vice versa if illuminated from HTL side. The PSC device under consideration is illuminated from the ETL side; therefore, only the impact of perovskite/ETL interface defect density (IDD) is considered.

**Figure 6:**
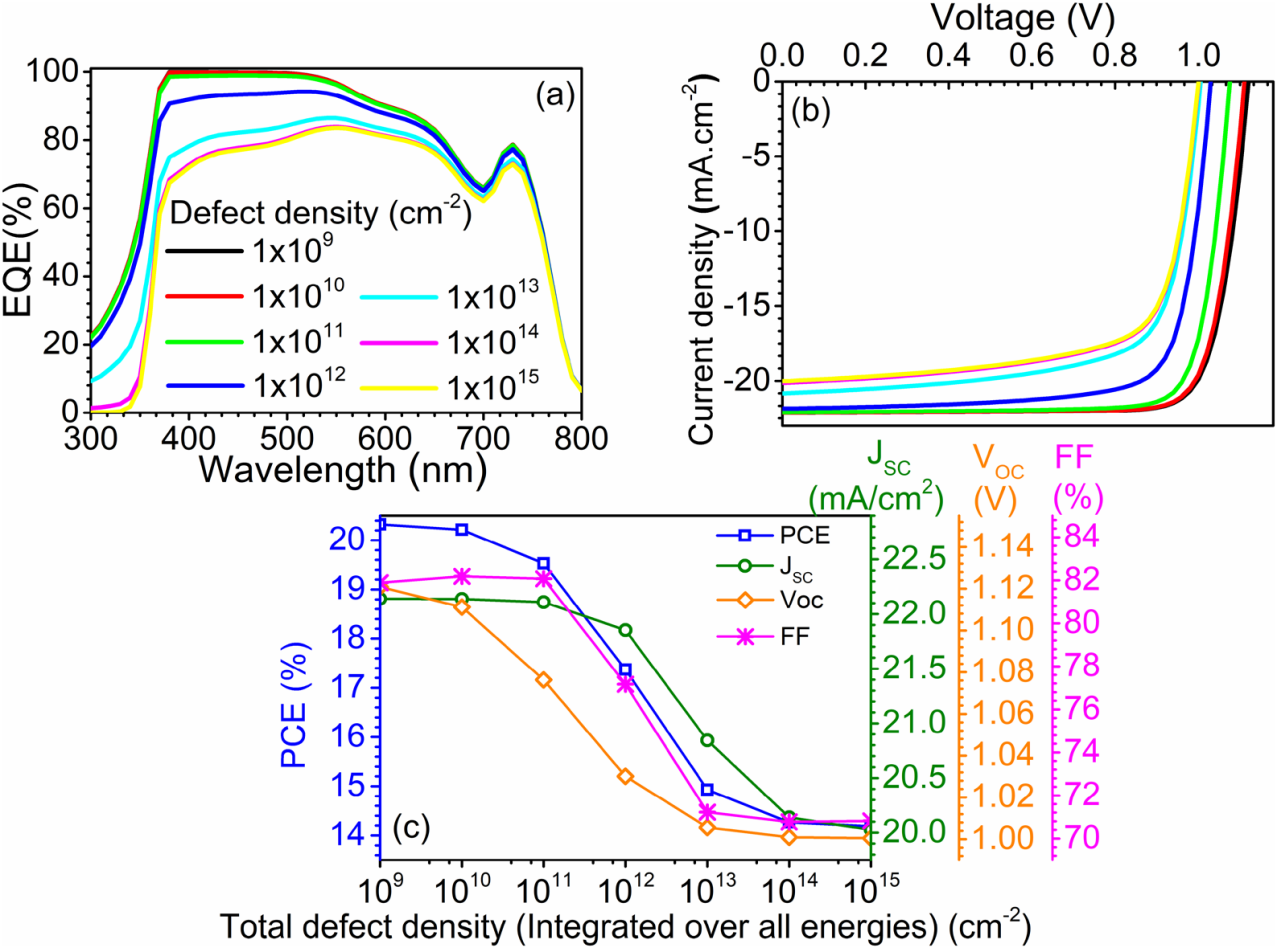
Impact of perovskite/ETL interface defect density (IDD) on (**a**) EQE, (**b**) illuminated J–V curve, and (**c**) PV parameters. Data reported in figures are obtained from the SCAPS-1D simulator.

The investigation depicts that IDD at the perovskite/ETL interface has a strong effect on the performance of the device. Here the IDD (integrated over all energies) is varied from 1 × 10^9^ cm^−3^ to 1 × 10^15^ cm^−3^ in seven equal steps in a logarithmic scale. The corresponding SRV for both electrons and holes is 10^1^ cm.s^−1^ (at lowest IDD) and 10^7^ cm.s^−1^ (at highest IDD). Figure 6(a), (b), and (c) represent the EQE, J–V curves, and PV parameters, respectively, at different IDD. Increasing the IDD from 1 × 10^9^ cm^−3^ to 1 × 10^15^ cm^−3^ reduces the EQE, particularly at the lower wavelengths, as shown in Fig. 6(a). This is owing to the fact the lower wavelength photon generates the carriers in perovskite near the perovskite/ETL interface, and elevated IDD resulted in enhanced recombination and lower collection probability. Reduction is EQE leads to the drop in J_SC_ from 22.14 mA cm^−2^ to 22.02 mA cm^−2^ (Fig. 6(b-c)). The V_OC_ also dropped to 1.00 V from 1.12 V, and the decrease is attributed to a reduction in carrier concentration due to interfacial recombination, which rapidly neutralizes the light generated carriers. Result also reveals a drop of 11.07% in FF, which was initially 81.88%, and dropped to 70.81% as the IDD increased to 1 × 10^15^ cm^−3^ from 1 × 10^9^ cm^−3^. Due to the reduction in J_SC_, V_OC_, and FF, the overall PCE of the device reduces from 20.32% to 14.18%, which unveils that IDD at the perovskite/ETL interface has a considerable effect on the device performance.

### Design of perovskite-PbS CQD tandem solar cell

**Figure 7:**
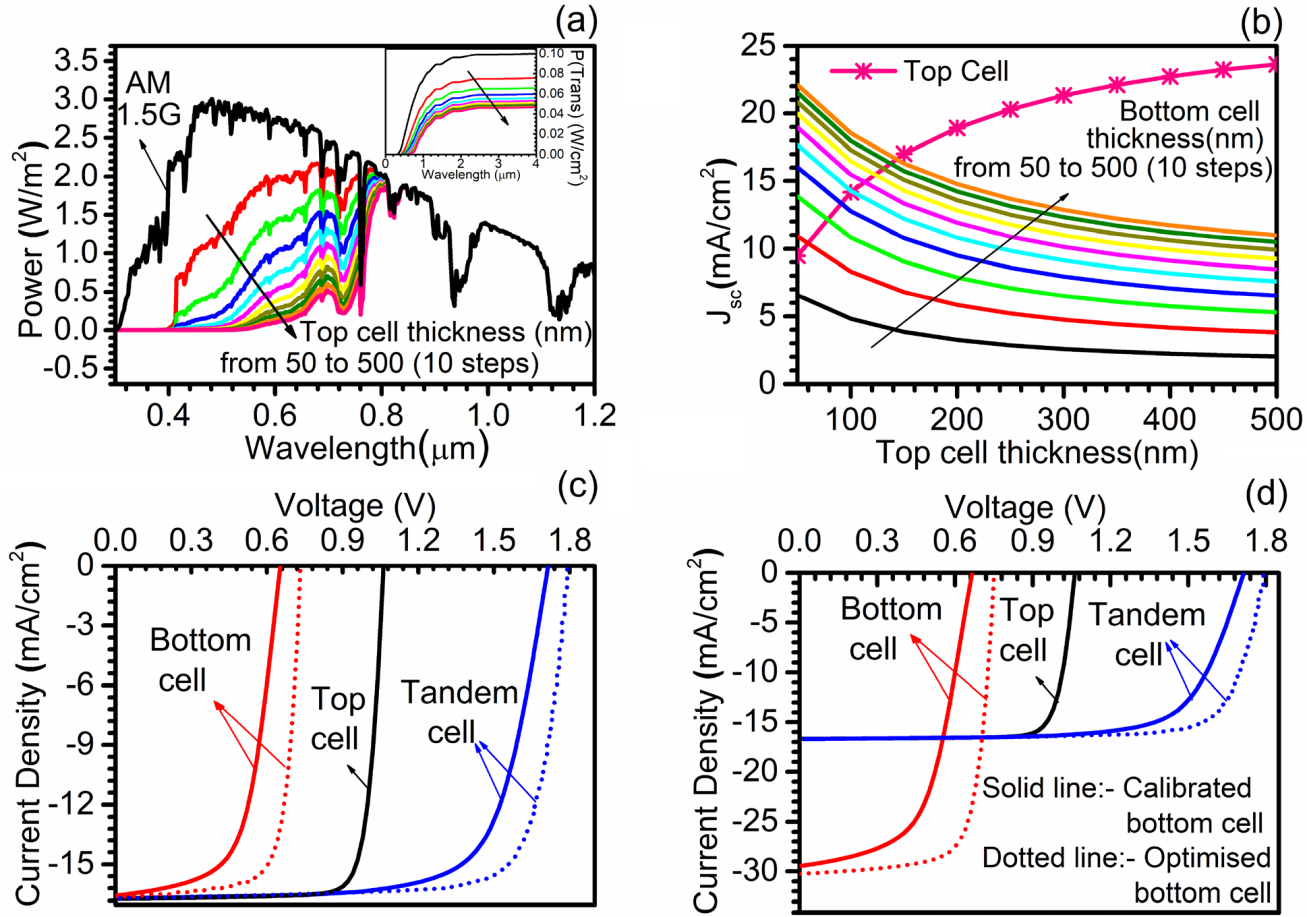
(**a**) Power transmitted by the top cell at different thickness along with standard AM1.5G spectrum, (**b**) current matching curve at the different top and calibrated bottom cell, (**c**) JV curve for standalone top cell, standalone bottom cell (calibrated and optimized), and tandem (with calibrated and optimized bottom subcell) solar cell and (**d**) JV curve for top cell and bottom cell (calibrated and optimized) under standalone (AM1.5G spectrum), the final tandem J–V curve with calibrated and optimized cell is also shown. Data reported in figures are obtained from the SCAPS-1D simulator.

In this section, a thorough study of the tandem structure formed by combining the perovskite-based top cell and PbS CQD-based bottom cell is reported. The perovskite top cell is identical to as optimized in the previous subsection whereas the calibrated PbS CQD bottom subcell is invoked from our previous publication ^45^. The methodology of tandem solar cell simulation has been mentioned in simulation methodology. In brief, the top cell has been illuminated with the standard AM1.5G, whereas the bottom cell is exposed to the filtered spectrum calculated by using absorption coefficients as well as thickness of the various materials used in the top cell. In total, ten (10) filtered spectrums by the top cell are obtained by varying the thickness of the perovskite absorber layer from 50 to 500 nm in the top subcell while keeping the other parameters intact. Figure 7(a) represents the computed filtered spectrum and associated transmitted power (P_trans_) at each thickness level. The reduction in the filtered transmitted power, particularly below the 800 nm wavelength range, is observed with the increase in the thickness of the perovskite layer. This is attributed to higher absorption in the top cell with the upturn in thickness, and it is worth noting that perovskite with only 50 nm thickness reduces the power level of standard AM1.5 spectrum from 0.1 W cm^−2^ to 0.076 W cm^−2^ in term of the filtered spectrum, inset of Fig. 7(a). Trends show higher transmitted photocurrent and low absorbed current in the top cell for low values of perovskite thicknesses, i.e., 50 nm. Raising the thickness to higher values, i.e., towards 500 nm, elevates the absorbed photocurrent and simultaneously reduces the transmitted photocurrent.

Two terminal monolithic tandem solar cell structure resembles the architecture of back-to-back connected or series-connected diodes in which the cell with minimum current in the constituent sub-cells limits the maximum current, i.e., tandem J_SC_ whereas the V_OC_ is the sum of the individual V_OC_ of the sub-cells. Mathematically, J_SC_ (tandem) ~ min {J_SC_ (top), J_SC_ (bottom)} in standalone configuration and V_OC_ (tandem) ~ V_OC_ (top) + V_OC_ (bottom). Hence, we have augmented the thicknesses of both the subcells so as to match maximum deliverable J_SC_ from the tandem solar cell, which is constrained by the limiting cell, i.e., top cell. In order to obtain maximum J_SC_ from the tandem device, the materials having different bandgaps should be adopted so that both of them can perform in their defined areas of the power spectrum. Apart from that, current matching point should be at such an appropriate thickness level that it should avoid parasitic absorption in the top cell and should not limit bottom cell performance as well. In order to achieve the same, the calculated the filtered power spectrum for ten (10) different thicknesses of the perovskite absorber (50 nm to 500 nm) is used to conceive the presence of the top cell with different perovskite thickness from 50 to 500 nm. These spectrum files are fed to the bottom sub-cell so as to measure its performance. Concurrently the thickness of PbS-TBAI of the bottom cell is also varied in 10 equal steps from 50 to 500 nm in order to calculate all the possible thickness value points where current matching could be possible. This effort resulted in the formation of current matching, i.e., J_SC_ matching curve as shown in Fig. 7(b) at various thickness of the top and bottom subcells. The result shows that the current matching is not observed for the thickness of both cells equivalent to 50 nm and for the perovskite thickness more than 150 nm. Whereas nine (9) intersecting points are observed for the perovskite thickness range 50 nm to 150 nm and PbS-TBAI thickness range 100 nm to 500 nm. Among them, the last current matching point has opted to deliver the maximum tandem J_SC_ of 16.62 mA cm^−2^ and the corresponding top and bottom subcell thickness 143 nm and 500 nm, respectively.

After identifying the corresponding top and bottom subcell thickness to get maximum J_SC_ from the tandem device, the top cell is re-simulated with thickness 143 nm under standard AM1.5G spectrum, and another filtered spectrum is constructed with said thickness. The same filtered spectrum has been used to illuminate the bottom subcell with a thickness of 500 nm to obtain the illuminated J–V of the bottom subcell under tandem configuration. The J–V curve for both top cell (143 nm) under AM1.5G spectrum and bottom cell (500 nm) under filtered spectrum is depicted in Fig. 7(c), which validates the equal current, i.e., current matching in both top and bottom subcells. Followed by current matching, the tandem J–V curve is constructed by adding the voltage (x-axis) at equal current points (y-axis) to construct the tandem J–V curve, which is shown alongside the individual J–V curve in Fig. 7(c). The solid line tandem curve shown in Fig. 7(c-d) is constructed using calibrated PbS-CQD bottom subcell having PbS-TBAI thickness of 500 nm and showed the tandem conversion efficiency of 20.57% with V_OC_ (1.714 V), J_SC_ (16.62 mA cm^−2^), and FF (72.3%).

It should be noted that the constructed tandem device did not contain the optimized bottom subcell in terms of doping density of PbS-EDT (HTL) and MZO (ETL). Therefore, to further enhance the conversion efficiency of the tandem device, the optimized doping 5 × 10^17^ cm^−3^ is used for both HTL and ETL layers in the bottom subcell. The detailed discussion related to the optimization of the doping level in the bottom subcell is reported in our previous work published elsewhere ^45^. In standalone configuration the calibrated bottom cell with thickness 500 nm showed 11.24% conversion efficiency with V_OC_ (666 mV), J_SC_ (29.64 mA cm^−2^) and FF (56.9%) whereas optimized bottom cell reflected 16.88% conversion efficiency with V_OC_ (752 mV), J_SC_ (30.77 mA cm^−2^) and FF (72.9%). The significant improvement in V_OC_ and FF anticipates the improvement in the conversion efficiency of the tandem device. However, a 1.13 mA cm^−2^ higher J_SC_ value would disturb the current matching criteria that are obtained with calibrated device. Therefore, under filtered spectrum by 143 nm top cell, the thickness of PbS-TBAI in the optimized device is adjusted to 470 nm in order to have the same current matching point, i.e., similar current as of top cell. This exercise led to the formation of the J–V curve for the optimized bottom subcell (shown in the dotted line) under the filtered spectrum by the top cell. Now it can be clearly validated through Fig. 7(c) that the J_SC_ of top cell and bottom cell for both calibrated as well as optimized device ties at the same point. This supports that another tandem curve could be constructed with an optimized bottom subcell without disturbing the current matching criteria by keeping this fact in mind that for current matching with 143 nm top subcell, the thickness of bottom subcell is 500 nm and 470 nm in for calibrated and optimized bottom subcell, respectively. The tandem device constructed with optimized bottom subcell reflected 23.36% conversion efficiency with 1.79 V (V_OC_), 16.67 mA cm^−2^ (J_SC_) and 78.3% (FF). The tandem device with optimized bottom subcell showed 13.6% higher conversion efficiency, and the improvement is credited to 4.4%, and 8.3% enhanced V_OC_ and FF, respectively, compared to the tandem device having calibrated bottom subcell. Further, standalone top and bottom subcell J–V curve with optimized thickness in respect to the tandem configuration is also reported in Fig. 7(d) along with tandem J–V curve and quantitative summary of the PV parameter for all the devices considered in this subsection are provided in Table 3. This reported thorough analysis of perovskite-PbS CQD tandem solar cell concludes a 23.36% efficient tandem design.

**Table 3:** Summary of the photovoltaic parameters of all the devices considered in this subsection.

Device		Thickness (nm)	J_SC_ (mA cm^−2^)	V_OC_ (V)	FF (%)	PCE (%)	Source
Perovskite top cell (standalone)		143*	16.68	1.061	82.4	14.60	This work
Experimental PbS-CQD bottom cell (standalone)		230	24.50	0.620	62.0	9.41	Zhang et al.^44^
Calibrated PbS-CQD bottom cell (standalone)		230	24.51	0.633	60.8	9.43	Pandey et al.^45^
Optimized PbS-CQD bottom cell (standalone)		230	25.07	0.730	74.3	13.59	Pandey et al.^45^
Calibrated PbS-CQD bottom cell	Standalone (AM1.5G)	500*	29.48	0.666	56.9	11.19	This work
	Under filtered spectrum		16.57	0.653	61.5	6.65	
Optimized PbS-CQD bottom cell	Standalone (AM1.5G)	500	30.61	0.752	72.9	16.80	
	Under filtered spectrum		16.97	0.735	74.4	9.27	
	Standalone (AM1.5G)	470*	30.24	0.751	73.2	16.63	
	Under filtered spectrum		16.62	0.733	74.5	9.07	
Perovskite/PbS-CQD tandem with calibrated bottom cell		143 (top) /500 (bottom)	16.62	1.714	72.3	20.57	
Perovskite/PbS-CQD tandem with optimized bottom cell		143 (top) /470(bottom)	16.67	1.790	78.3	23.36	

*Thickness for current matching.

## Conclusion

This work concludes a 23.36% efficient two-terminal monolithic perovskite-PbS CQD tandem solar cell design thorough comprehensive device simulations. Before evaluating the tandem design, a detailed standalone analysis of perovskite top cell is carried out in terms of variation in optical properties (in accordance with experimental data), absorber layer thickness, and absorber layer/transport layer interface defect density so as to optimize the standalone performance of the top cell. Further, the filtered spectrum and associated integrated power transmitted by optimized top cell at different perovskite layer thicknesses are obtained for the tandem simulation. The same filtered spectrum file is used to simulate the calibrated and optimized PbS-CQD based bottom subcell to get the current matching conditions. This has been done to get the maximum deliverable J_SC_ of the tandem device by varying the thickness of the absorber layer in both the top and bottom subcell. Top and bottom subcell reflected 14.60% and 9.07% conversion efficiency with approximately equal current 16.68 mA cm^−2^ (top) and 16.62 mA cm^−2^ (bottom) under tandem configuration with an optimized thickness of 143 nm (top cell) and 470 nm (bottom cell), where the top cell is simulated under AM1.5G spectrum, and bottom cell is stimulated by the spectrum filtered by 143 nm thick top cell. Distinctly measured J–V curves of the top (under AM1.5G) and bottom subcell (under filtered spectrum) at equal current are summed together in series. In other words, the voltages are added at equal current to generate tandem J–V characteristics. This work concludes a tandem design which could deliver 23.36% conversion efficiency with 1.79 V (V_OC_), 16.67 mA cm^−2^ (J_SC_) and 78.3% (FF).

Further, in the current manuscript, a maximum J_SC_ of 22.14 mA cm^−2^ and 30.61 mA cm^−2^ has been achieved in the standalone simulation of top and bottom subcells, respectively, which clearly shows that the perovskite top cell with J_SC_ of 22.14 mA cm^−2^ needs to act as a limiting cell for the tandem J_SC_. Therefore, a tandem design that can deliver a J_SC_ of 22.14 mA cm^−2^ would be considered as an optimum design; however, the constructed tandem device showed the tandem J_SC_ of 16.67 mA cm^−2^ which is much lower compared to the limiting top cell. Therefore, the main limiting cell in the proposed design is not the top cell but the bottom cell as it is not able to maintain the J_SC_ close to the limiting cell under tandem configuration (filtered spectrum). The same can be validated through the current matching curve provided in the manuscript. Therefore, it is required to have a PbS-CQD solar cell with a higher J_SC_ value in standalone configuration so that the bottom cell can maintain a significant level of J_SC_ when placed underneath the top cell, i.e., under filtered spectrum to match the J_SC_ of standalone top cell. Therefore, there is a need to design a PbS-CQD solar cell with higher J_SC_ values followed by the optimization of the top cell in the future to lift the conversion efficiency of the proposed design beyond the standalone perovskite solar cells. In addition, the detailed study of the tandem design reported in the current study would open the path for the development of high-efficiency, low-cost perovskite-PbS CQD tandem solar cells in the future. The influence of the physical presence of tunnel recombination junction can be done in the future.

## Data Availability

The data (problem definition file of top and bottom cell, filtered spectrum, and script for tandem J–V curve) that support the findings of this study shall be made available from the corresponding author upon reasonable request.
